# Longitudinal Relation between Family Socio-Economic Status and Problem Behaviors in Chinese Children: The Roles of Sense of Coherence and Maternal Warmth

**DOI:** 10.3390/bs13040291

**Published:** 2023-03-29

**Authors:** Bibo Mo, Rui Fu, Xiaoshi Liu, Gangmin Xu, Junsheng Liu, Dan Li

**Affiliations:** 1 Department of Psychology, Shanghai Normal University, Shanghai 200030, China; 2 Center for Violence Prevention at Children’s Hospital of Philadelphia, Philadelphia, PA 19146, USA; 3 Department of Psychology, Yangzhou University, Yangzhou 225002, China; 4 School of Psychology and Cognitive Science, East China Normal University, Shanghai 200333, China

**Keywords:** family socioeconomic status (SES), externalizing problems, internalizing problems, sense of coherence, maternal warmth

## Abstract

Literature has well-documented the relation of family socio-economic status (SES) to children’s problem behaviors, yet the complex mechanisms underlying the relation are not well understood. Therefore, the primary goal of this one-year longitudinal study was to explore the mediating role of children’s sense of coherence and the moderating role of perceived maternal warmth in the association between family SES and externalizing and internalizing problems in Chinese children. The sample consisted of 913 children (493 boys; M_age_ = 11.50 years, SD = 1.04) in fourth to sixth grades in an urban area in mainland China. Data were obtained from multiple sources, including child self-reports, parental reports, and teacher ratings. The results indicated that children’s sense of coherence mediated the association between family SES and internalizing problem behaviors, but not externalizing problem behaviors. This mediating role was also moderated by maternal warmth and specifically, family SES was negatively associated with internalizing problem behaviors via the sense of coherence for children who perceived high maternal warmth. Generally, these results highlighted the possible roles of a sense of coherence and maternal warmth in the longitudinal implications of family SES for Chinese children’s internalizing problems.

## 1. Introduction

Research on children’s and adolescents’ social development has been largely focused on problem behaviors, including externalizing and internalizing problem behaviors [1,2,3]. It is shown that externalizing problem behaviors, such as aggressive, hyperactive, and disruptive behaviors, positively predict peer rejection and low academic performance [4,5]. Similarly, it has also been found that internalizing problem behaviors (e.g., anxiety, depression) positively predict sleep problems and peer victimization [6,7]. According to the social causation theory [8,9], the shortage of material resources in the family is likely to expose children to stress, which in turn leads to subsequent problem behaviors, suggesting economic difficulties as a stressor impacting child development. This proposition has been supported by several longitudinal studies [10,11] that family socioeconomic disadvantage leads to long-term problem behavior.

However, our understanding of the relationship between socioeconomic status (SES) and problem behaviors has been limited to individual-oriented, Western societies, leaving this issue largely understudied in group-oriented societies, such as China. A few studies examining the mechanisms underlying the association between family SES and academic achievement in China have suggested the roles of family process factors in mediating and moderating the association [12]. Yet little research has examined the underlying mechanism in the relation between SES and other adjustment domains, especially problem behaviors that contribute to the comorbidities of adjustment difficulties [13]. As such, additional work is needed more specifically on factors that help to explain or modify the relation between family SES and problem behaviors in Chinese children.

Previous studies have demonstrated that a strong sense of coherence as a positive personal characteristic [14] jointly with maternal warmth as an optimal contextual factor [15] buffer individuals against maladjustment development. However, their roles in protecting children residing in families with limited resources from problems in Chinese children remain understudied. To address the gaps, the present study aimed to explore the relations between family SES and externalizing and internalizing problem behaviors and the role of a sense of coherence and maternal warmth in mediating and moderating the relations in Chinese children.

### 1.1. Family SES and Children’s Problem Behaviors in the Chinese Context

Problem behaviors, including both internalizing and externalizing problem behaviors, are prevalent among Chinese children [16]. Internalizing problem behaviors are defined as a problematic syndrome that includes depression, anxiety, as well as withdrawal behavior, and so forth [17]. Externalizing problem behaviors refer to a group of behaviors that reflect the negative reactions of children to the external environment, including aggressive, hyperactive, and disruptive behaviors [18,19]. In the Chinese context where prosociality and behavioral appropriateness are emphasized [20], externalizing behavior problems are perceived by others as indicating low self-control, selfish or anti-collective and have thus been robustly associated with socioemotional and school maladjustment, such as low academic achievement and life dissatisfaction [21,22,23]. At the same time, children in China have been found to display high levels of affect disturbances and other internalizing problems [16], which strongly contribute to child maladjustment in the Chinese context [24,25]. Given the increased attention on problem behaviors, particularly internalizing problems, from professionals and the public in recent years [26], it is necessary to investigate stressors that are associated with such behaviors and potential factors that buffer the associations among Chinese children.

SES is a measurement index reflecting a person’s status or prestige relative to others [27]. Generally speaking, family SES encompasses parental education, family income, and occupational status [8,28]. Specifically, parental education refers to skills and abilities necessary for individuals to be productive members of society (human capital), family income represents a family’s ability to purchase material resources (financial capital), and occupational status reflects one’s social standing in the occupational hierarchy and social network resources (social capital) [8,29].

As posited in Bronfenbrenner’s ecological systems theory [30], family SES is an important factor in the microsystem, the most immediate environment in which children live. It determines a family’s level of access to and control over economic and social resources that contribute to children’s well-being [31]. This theoretical argument has been endorsed in many empirical studies which demonstrate the critical implications of family SES for children’s educational achievement, and physical and psychological health [9,13]. These implications seem to be related to adequate resources such as nutrition, access to good health care, and cognitively stimulating materials and experiences, which facilitate the development of children’s well-being [8].

Compared to Western studies in which the role of family SES in predicting problem behaviors has been widely endorsed [32,33], this predicting role of family SES has been less studied in China as the majority has focused on its prediction of Chinese children’s academic achievement [34]. Considering that the importance of socioemotional well-being has been increasingly recognized in contemporary China [35,36], it is warranted to better understand the association between family SES and children’s internalizing problems. Furthermore, using a longitudinal approach would allow us to explore the role of family SES in contributing to the change in internalizing problems.

### 1.2. The Mediating Role of Sense of Coherence

Sense of coherence refers to “a global orientation that expresses the extent to which one has a pervasive, enduring though dynamic feeling of confidence” ([37] p.19). Antonovsky [37] pointed out the three components of sense of coherence, encompassing comprehensibility (the extent to which an individual perceives the stimuli from internal and external environments to be structured, predictable, and interpretable); manageability (the extent to which an individual use resources to address or react to stimuli); and meaningfulness (the extent to which an individual considers the stimuli worth investing their time and efforts to address). It is viewed as a dispositional orientation that helps individuals sustain and promote their physical and mental health [37,38]. According to the Salutogenic model, generalized resistance resources foster an individual’s ability to effectively respond to stress which in turn enhances an individual’s sense of coherence [37,39]. In other words, when chronic generalized resistance resources are integrated into an individual’s life situation, these resistance resources are considered the primary determinants of the strength of the individual’s sense of coherence level [40].

As a type of chronic, generalized resistance resources, family SES, is strongly associated with family coping resources. These resources not only denote the materials and strategies within the family that the person can rely on to manage stressful events, but also provide the foundation for the three components (i.e., comprehensibility, manageability, and meaningfulness) of sense of coherence [40,41]. Individuals with higher levels of sense of coherence tend to view stressful events as predictable, explainable, and meaningful [38]. This positive orientation when coping with life stressors is evidenced to lead to effective coping [42,43] and positive, future-focused emotions [44]. These positive coping responses are likely to be associated with fewer problem behaviors, such as substance use, depression, and anxiety [45,46]. Taken together, family SES may indirectly contribute to children’s problem behaviors via a sense of coherence, which may play a mediating role in the underlying influencing mechanism. However, this speculation has not been addressed in the literature.

As posited in the Salutogenic model, the contribution of a sense of coherence to promoting children’s overall adjustment is viewed as universal [37,47] and empirical findings in many Western societies (e.g., Australia, Belgium, Brazil; [2]) have lent support to this postulation [46,48]. However, compared to Western research, studies on the role of the sense of coherence in group-oriented, non-Western societies, namely, China, are still lacking. In Chinese culture, the need for individuals to develop the ability to suppress emotions and impulses is highly emphasized, and over-expression of impulsive behaviors is viewed as a sign of immaturity [49]. Self-control plays a pivotal role in Chinese children’s development of social competence, which is manifested in curbing one’s anger or aggressive impulses in face of conflicts [49,50]. Moreover, self-control is regarded as a dimension of manageability, one core component of sense of coherence [51]. Children with a high sense of coherence are self-controlled which helps them monitor and modulate their negative emotional reactivity and demonstrate other effective coping responses [43]. In addition, children with a high sense of coherence tend to have a pervasive feeling of self-confidence which is increasingly valued in contemporary China, particularly in urban areas with increased competitiveness [52,53].

The social change in competitive urban China has manifested in changes in values that facilitate the development of cooperation and interdependence to those that facilitate the development of individual autonomy and independence [49]. Given the changes in cultural values, it is critical to examine the role of sense of coherence in predicting Chinese children’s adjustment. Furthermore, as China’s economy continues to grow, household income inequality has widened and been accompanied by financial distress in low SES families [54,55]. It is clear that family financial stress is positively associated with problem behaviors in China [56]. Therefore, the possible mediating role of sense of coherence in linking family SES and Chinese children’s adjustment is worth examining. It is possible that high family SES would be associated with increased levels of sense of coherence which is in turn related to fewer problem behaviors, as an important indicator of adjustment in Chinese children.

### 1.3. The Moderating Role of Maternal Warmth

Although the literature has documented that family SES is, in general, positively associated with sense of coherence and children’s positive development [57], not all children with abundant resources exhibit high levels of sense of coherence and adjustment. There is some research indicating that the impact of family SES on children may be enhanced or weakened by contextual factors (e.g., living regions; [58]). As suggested in the resource-potentiating model [59,60], positive conditions may help enhance individuals’ resources and promote adaptive development whereas adverse conditions may suppress adaptive development. According to this model, high maternal warmth may elevate the advantages of higher family SES as revealed in a stronger sense of coherence and/or fewer problem behaviors yet such advantages are likely less or not pronounced when maternal warmth is low. From a different perspective, as argued in the stress model [61], unfavorable social conditions may be an exacerbating factor, making children particularly vulnerable to risk and associated behavioral problems. On the contrary, positive conditions may function to buffer against the impact of stress on children. Specifically, in low SES families where there are limited financial resources, high maternal warmth may create a supportive familial climate for children [62,63] and provide them with consistently meaningful and coherent life experiences that are conducive to the development of the sense of coherence [64]. As such, these supportive provisions may enable the child to view stressful situations as comprehensible and manageable and to respond to the situations constructively, for example, turning to family for help rather than acting out or internalizing negative emotions.

Considering the possible role of maternal warmth in moderating the associations between family SES and sense of coherence and problem behaviors, the pathway of family SES to problem behaviors via the sense of coherence is likely to vary by the levels of maternal warmth. Specifically, this mediation effect may be weaker for children who experienced low levels of maternal warmth than for those who experienced high levels of maternal warmth. An orderly and supportive rather than chaotic family atmosphere, characterizing high levels of maternal warmth provide children with constructive modeling behaviors when coping with stressful events in the family that help them feel more in control and less anxious when facing difficulties in academic subjects or peer relationships [65,66]. Also, maternal warmth may represent a social and emotional resource that affords children to feel secure and confident when exploring their physical and social environments [67]. A strong sense of security and confidence is conducive to the growth of consistent emotional connectedness in social relationships which is evidenced in an enhanced level of sense of coherence [41,65,68]. In addition, high maternal warmth may enhance the functional meaning of sense of coherence as a mediator that links family SES and problem behaviors. That is, when mothers are affective, caring and responsive, high family SES is likely to provide children with more instrumental (e.g., rewarding the child for completing a tough school assignment) and emotional support (e.g., making the child feel valued and loved) that makes the mediating role of their sense of coherence more pronounced. In comparison, this mediating effect of a sense of coherence might be lessened in children with low levels of maternal warmth because the advantages of high family SES might be thus limited to materialistic, instrumental provisions. In other words, the mediating process of children’s sense of coherence in linking family SES and problem behaviors over time may depend on the levels of their perceived maternal warmth.

### 1.4. The Present Study

**Figure 1:**
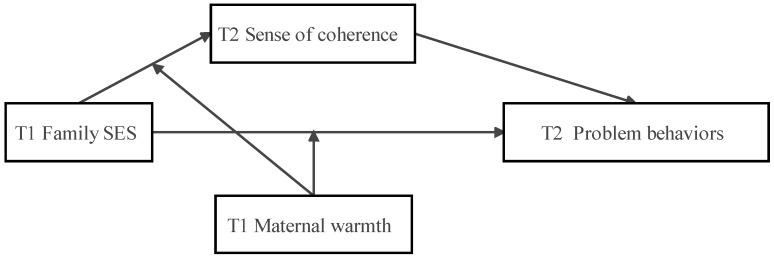
The Moderated Mediation Model. Note. T1 = Time 1; T2 = Time 2.

As suggested in Preacher (2015) [69] and Fitzmaurice et al. (2011) [70], the longitudinal moderated mediation technique is conducive to assessing whether an indirect effect over time is conditional on the values of a moderating variable. Further, this technique allows causal inferences about the mediation process. This one-year longitudinal study aimed to explore the role of sense of coherence in mediating the relations between family SES and Chinese children’s problem behaviors (i.e., externalizing and internalizing problems) and the moderating effects of maternal warmth on the associations between family SES and sense of coherence and problem behaviors over time, while controlling for the initial levels of sense of coherence and problem behaviors. As illustrated in the conceptual model in Figure 1, we examined the following hypotheses: (1) T1 family SES would negatively predict T2 problem behaviors; (2) T2 sense of coherence would mediate the association between T1 family SES and T2 problem behaviors; (3) T1 maternal warmth would moderate the associations between T1 family SES and T2 sense of coherence; (4) T1 maternal warmth would moderate the relationship between T1 family SES and T2 problem behaviors; and (5) T1 maternal warmth would moderate the mediating effect of T2 sense of coherence underlying the association between T1 family SES and T2 problem behaviors. Additionally, given the relatively short duration between T1 and T2 and prior findings of the stability of the sense of coherence [71], we hypothesized that there would be no difference in the sense of coherence across time.

## 2. Methods

### 2.1. Participants

Participants in the study included 913 4th to 6th grade children (493 boys; M_age_ = 11.50 years, SD = 1.04) from four randomly selected regular public schools in Shanghai, China. There were 17.01% of the children from low SES families with an average yearly family income being approximately 48,000 yuan (6941 USD). Of the mothers and fathers, 12.5% and 6.9% had an elementary school, 33.2%, and 33.2% had a junior high school, 18.2% and 24.3% had a senior high school degree, and 36.2% and 35.6% had a college degree or higher education. Of the children, 65.84% were only children, and the others had one or more siblings. A year later (Time 2), 673 students participated in the follow-up study. There were no significant differences between students who participated in the follow-up study and those who did not, in Time 1 sense of coherence, F (1, 857) = 1.18, *p* = 0.28, on Time 1 externalizing problem behaviors, F (1, 899) = 0.05, *p* = 0.82, and on Time 1 internalizing problem behaviors, F (1, 899) = 0.45, *p* = 0.50.

### 2.2. Measures

#### 2.2.1. Family SES

Given that the assessment of occupational status is often highly subjective [27], there is no consensus on the classification of occupations in China as people of the same occupation have large gaps in income and education levels [72]. Therefore, only parental education level and family income were used as indicators of SES [73,74]. Parents’ self-reported monthly income was used (e.g., wages, bonuses) as an indicator of family income, which was divided into eight categories, below 500 yuan (74.7 USD), 500–1000 yuan (74.7–149.4 USD), 1000–2000 yuan (149.4–298.8 USD), 2000–3000 yuan (298.8–448.2 USD), 3000–5000 yuan (448.2–747 USD), 5000–10,000 yuan (747–1494 USD), 10,000–20,000 yuan (1494–2988 USD), and above 20,000 yuan (2988 USD). The monthly incomes of both parents were summed up to represent family income. Parental education ratings for both parents were coded using a 4-point scale indicating “1 = elementary school and below”, “2 = junior high school”, “3 = high school or technical secondary school”, and “4 = college and above”. Following the recommended approach by Kraus and colleagues [75,76], an index of family SES was formed by totaling the standardized scores for parental education and family income. (i.e., Family SES = Z_paternal income_ + Z_maternal income_ + Z_paternal education_ + Z_maternal education_, with higher scores indicative of greater family SES. In this study, internal reliability of the measure (Cronbach’s α) was 0.85.

#### 2.2.2. Sense of Coherence

The adapted version of the Children’s Sense of Coherence Scale [77] that consisted of 12 items was used and example items are “If I want something, I believe I can get it”, and “(reverse coded) I think what I do every day is meaningless”). Students were asked to respond to each item, on a 4-point scale, ranging from 1 (never) to 4 (always). Confirmatory factor analysis showed good model fit at both time points, χ^2^/df = 4.80 and 4.34, RMSEAs = 0.07, SRMRs = 0.06, CFIs = 0.93, TLIs = 0.91, respectively. As suggested by Liu et al. [77] and Antonovsky [78], the average score of all items was computed, with higher scores suggesting higher levels of self-perceived sense of coherence. The internal reliabilities (Cronbach’s α) were 0.75 and 0.79 at Times 1 and 2, respectively, in this study.

#### 2.2.3. Problem Behaviors

The head teacher in each class was requested to complete a 5-item measure of externalizing problems (e.g., “Disruptive in class”) and a 9-item measure of internalizing problems (e.g., “Anxious, worried”), adapted from the Teacher-Child Rating Scale (T–CRS; [79]). The teacher rated, on a 5-point scale, ranging from 1 (not at all) to 5 (very well), how well each of the items described the student. Consistent with the procedure used in previous studies [80], the teacher-rating scores were standardized within the class to adjust for the teacher’s response style and to allow for appropriate comparisons. Previous studies have suggested that it is a reliable and valid measure in Chinese children [80,81]. The results of the confirmatory factor analysis suggested good-fitting models for externalizing problems at Times 1 and 2, χ^2^/df = 4.61 and 4.87, RMSEAs = 0.06 and 0.08, SRMRs = 0.02, CFIs = 0.99 and 0.98, TLIs = 0.98 and 0.96, respectively, and for internalizing problems at Times 1 and 2, χ^2^/df = 4.73 and 3.33, RMSEAs = 0.06, SRMRs = 0.04, CFIs = 0.94, TLIs = 0.92 and 0.91, respectively. In this study, the internal reliabilities were 0.81 and 0.79 for externalizing problems at Times 1 and 2, and 0.75 and 0.71 for internalizing problems at Times 1 and 2.

#### 2.2.4. Maternal Warmth

Maternal warmth was assessed using an adapted Chinese version of the Child Rearing Practices Report (CRPR; [82]). Participating students were asked to rate four items (e.g., “When I feel uneasy or scared, my mother gives me comfort and understanding”), on a 5-point scale, ranging from 1 (not at all true) to 5 (always true). This measure has been shown to be reliable and valid in the Chinese context [83]. Factor confirmatory analysis showed that the one-factor model was acceptable (χ^2^/df = 1.79, RMSEA = 0.03, SRMR = 0.004, CFI = 0.999, TLI = 0.997). In this study, the internal reliability was 0.83.

### 2.3. Procedures

This study obtained data from multiple sources, including parental reports, child self-reports, and teacher ratings, in order to reduce common method biases [84]. Written assent was obtained from all participating children and written consent was obtained from their parents through the school. Self-report measures of sense of coherence and maternal warmth were administered during class time on a school day. Teachers were asked to complete a rating scale for each participant concerning his or her externalizing and internalizing problems. Extensive explanations were provided to participants during data collection. The administration of all measures was carried out by a group of psychology teachers and graduate students in China. Data were collected in November 2013 (Time 1) and 2014 (Time 2). This study followed APA ethical guidelines and was approved by the institutional review board of the first author’s university.

### 2.4. Statistical Analysis

Descriptive statistics and intercorrelations among the study variables were conducted using SPSS (version 22.0). Then, moderated mediating modeling was conducted in Mplus (version 8.3). Little’s MCAR test [85] was significant, χ^2^(139) = 191.26, *p* = 0.002. However, the χ^2^/df was 1.38 (i.e., less than the suggested cutoff value of 2; [86]), indicating that the pattern of missing data is not substantially different from the random pattern [87]. Therefore, we employed the full information maximum likelihood (FIML; [88]) to handle missing data. The model fit was evaluated by the comparative fit index (CFI), the Tucker-Lewis index (TLI), the root mean square error of approximation (RMSEA), and the standardized root mean square residual (SRMR). Acceptable and good fit was indicated by RMSEA values below 0.08 and 0.05 and CFI values greater than 0.90 and 0.95, respectively [89,90].

## 3. Results

### 3.1. Descriptive Data

Means and standard deviations for and intercorrelations among the study variables are presented in Table 1. Results of repeated measures ANOVA showed no difference in sense of coherence across time, F(1, 632) = 0.02, Wilks’ λ = 1, *p* = 0.89. T1 family SES, T1 maternal warmth, and T1 and T2 sense of coherence were positively intercorrelated. Also, there were concurrent and longitudinal negative correlations between the sense of coherence and, externalizing problem behaviors and internalizing problem behaviors.

### 3.2. The Mediating Effect of Sense of Coherence

Following the suggestions of Hayes and colleagues [91,92], we estimated indirect effects in mediation analyses using the bias-corrected bootstrapping method with 2000 bootstrap samples. This method produces bootstrapped confidence intervals, with mediation occurring when the indirect effect is significant and its 95% bias-corrected confidence interval does not contain zero [93,94]. The mediation model was good fitting, χ^2^/df = 2.59, RMSEA = 0.05, SRMR = 0.03, CFI = 0.97, TLI = 0.91. The results in Table 2 showed that the direct and total effects of T1 family SES on T2 externalizing and internalizing problems were nonsignificant, controlling for child gender and age, and T1 externalizing and internalizing problems. There was a significant indirect effect of T1 family SES on T2 internalizing problems via T2 sense of coherence, whereas the indirect effect of T1 family SES on T2 externalizing problems via T2 sense of coherence was nonsignificant, suggesting the mediating role of T2 sense of coherence in the pathway of T1 family SES and T2 internalizing problems.

### 3.3. The Moderating Effect of Maternal Warmth

Because the mediation of T2 sense of coherence between T1 family SES and T2 internalizing problems was significant, the moderated effect of T1 maternal warmth on this mediation was examined, controlling for child gender and age. The moderated mediation model suggested good model fit, χ^2^/*df* = 1.33, RMSEA = 0.03, SRMR = 0.01, CFI = 0.99, TLI = 0.98. Controlling for T1 internalizing problems, T1 sense of coherence, and child gender and age, T1 maternal warmth moderated the mediating effect of T2 sense of coherence on the association between T1 family SES and T2 internalizing behavior problems, β = −0.01, *SE* = 0.005, *t* = −2.09, *p* = 0.037, 95% CI = (−0.021, −0.002).

**Figure 2:**
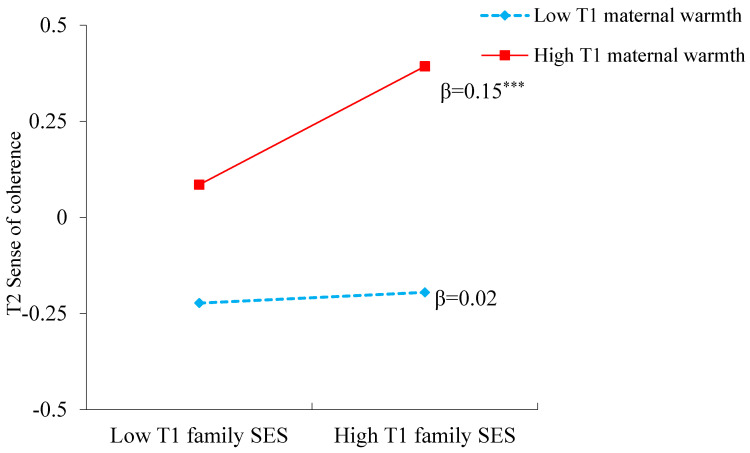
Simple Slope for Interaction Between Family SES and Maternal Warmth in Predicting Sense of Coherence. *Note.* T1 = Time 1; T2 = Time 2. *** *p* < 0.001.

In addition, the interaction of T1 maternal warmth and family SES on T2 sense of coherence was significant, β = 0.07, *SE* = 0.02, *t* = 3.12, *p* = 0.002, yet this interaction on internalizing problems was not significant, β = 0.03, *SE* = 0.03, *t* = 1.06, *p* = 0.29. Further simple slope effects were examined on T1 family SES at high and low values (1 *SD* above and 1 *SD* below the mean) of maternal warmth. As shown in Figure 2, for children with high T1 maternal warmth, T1 family SES was significantly and positively associated with T2 sense of coherence, β = 0.15, *SE* = 0.03, *t* = 4.62, *p* < 0.001, whereas this association was nonsignificant for children with low T1 maternal warmth, β = 0.02, *SE* = 0.03, *t* = 0.46, *p* = 0.65. Moreover, when T1 maternal warmth was high, T1 family SES was negatively associated with T2 internalizing problem behaviors via T2 sense of coherence, β = −0.02, *SE* = 0.01, *t* = −2.29, *p* = 0.022, 95% CI = (−0.037, −0.005), whereas this mediating effect of T2 sense of coherence was not significant when T1 maternal warmth was low, β = −0.002, *SE* = 0.004, *t* = −0.42, *p* = 0.672, 95% CI = (−0.011, 0.006).

## 4. Discussions

Research on the relation between family SES and developmental outcomes in Chinese children has mainly focused on the outcome of academic achievement [12,95] while the relation between family SES and adjustment in other domains remain less studied. Considering that the importance of individual socioemotional well-being has been increasingly recognized in contemporary China [35,36], As such, additional research is needed to better understand the implications of family SES for child outcomes such as externalizing and internalizing problems that contribute to the comorbidities of behavioral difficulties. Moreover, factors that may help explain and alter family SES disparities in youth outcomes are not well understood. The present study sought to fill the gaps by examining the roles of a sense of coherence and maternal warmth in explaining the association between family SES and problem behaviors among Chinese children. Results provided supporting evidence that children’s sense of coherence served to mediate the association between family SES and internalizing problems over time and that this mediating role was particularly pronounced in children with high maternal warmth.

### 4.1. The Mediating Role of Sense of Coherence

Contrary to Hypothesis 1, family SES was not significantly associated with externalizing and internalizing problem behaviors over time. This may suggest that the longitudinal relation between family SES and problem behaviors may be curvilinear rather than linear, as suggested in recent meta-analytic work [32]. That is, children from high and low SES backgrounds may show comparable levels of problem behaviors, possibly attributed to different pathways of family SES leading to similar behavioral problems [96]. Nevertheless, these arguments should be examined using multi-wave (at least three-time points) longitudinal data in order to obtain a better understanding of the developmental patterns of the impact of family SES. Furthermore, the present study found little change in sense of coherence over time, which is consistent with previous research [71]. This may be due to a sense of coherence that is assumed to be a stable character orientation [97].

The indirect effect of a sense of coherence was significant in linking family SES and children’s internalizing problems later on. This finding partially supported Hypothesis 2 and lends support to Korous and colleagues’ [98] call for more studies that identify specific pathways that elucidate the impact of family SES on child development. According to the Salutogenic model [37], family SES is a major generalized resistance resource that provides consistent, load-balanced life experiences to children [40,99]. These positive and rich experiences are likely to foster the child’s ability to effectively cope with stressful events, which is central to one’s sense of coherence. Having a strong sense of coherence enables individuals to perceive daily life experiences, in general, to be manageable, to derive meaning from difficult events, and to cope with these events positively [37,46], which in turn lower the likelihood of internalizing frustration, anxiety, and other negative emotions. This result extends prior findings [40,41] in suggesting sense of coherence as a mediator underlying the association between family SES and later adjustment outcomes in Chinese children.

Moreover, this positive role of a sense of coherence suggests that this orientation is highly relevant for and conducive to self-regulation and self-confidence, two cultural values that are strongly valued in contemporary urban China [20,37]. On the other hand, sense of coherence did not mediate the longitudinal association between family SES and Chinese children’s externalizing problems. It may be that externalizing problems, such as aggressive behavior, is strictly prohibited in China in which maintaining social harmony is one primary socialization goal [20,60]. Therefore, social and contextual factors such as peer rejection may be more powerful in explaining the association between family SES and externalizing problems than individuals’ behavioral dispositions, such as a sense of coherence. Nevertheless, this argument is highly speculative and future studies need to validate the current finding.

### 4.2. The Moderating Role of Maternal Warmth

As expected in Hypothesis 3, family SES was positively associated with a later sense of coherence more strongly for children with high maternal warmth as compared to those with low maternal warmth. This finding indicated the important role of maternal warmth in promoting the advantages of high family SES, lending support to the resource-potentiating model [59] which posits that favorable social conditions facilitate the adaptive development of individuals who already have the advantage. As an index of positive parenting, maternal warmth provides children with emotional closeness and optimal mother-child interaction, which are the core contextual factors in the development of children’s sense of coherence [40,100,101]. The result further indicated that family SES and maternal warmth, each as a generalized resistance resource, individually and jointly contribute to promoting a sense of coherence. It is necessary for future researchers to pay attention to the possible interactive effects of other generalized resistance resources (e.g., self-efficacy and peer support) on the sense of coherence [40,46,102]. On the other hand, the current finding did not support Hypothesis 4. This may be attributed to the relationships between family SES and problem behaviors being non-linear [32]. In other words, the direction of the association between family SES and problem behaviors may fluctuate. Researchers have found that while low family SES may not provide adequate resources for children to curb problem behaviors [8,28], children from affluent families may also reactively engage in high levels of problem behaviors because they are subjected to pressure stemming from high parental expectations [103,104]. Therefore, the moderating role of maternal warmth may depend on the nonlinear contribution of family SES to problem behaviors, which cannot be examined in the present, two-wave longitudinal design. In addition, the nonsignificant role of maternal warmth as a moderator may suggest that the relationship between family SES and child adjustment, linear or nonlinear, is best explained by some combination of individual and contextual factors [33] rather than individual factors solely (sense of coherence in the current study). Future studies should continue exploring this issue.

More importantly, maternal warmth was found to moderate the mediating effect of sense of coherence on the longitudinal association between family SES and internalizing problems. Specifically, the mediating role of a sense of coherence was significant for children with high maternal warmth but not for others with low maternal warmth. High levels of perceived maternal warmth foster a strong parent-child emotional connection as a type of social-emotional resource that is pivotal for the steady growth of meaningfulness, comprehensibility, and manageability [37,97]. In other words, those children who live in a family environment abundant with attentiveness and respect are likely to feel in control of their lives and consider most school tasks and participation in social activities as meaningful and worthy of investing their efforts in, suggesting that high maternal warmth may strengthen the contribution of the sense of coherence as a mediator. In contrast, low maternal warmth appears to weaken the mediating function of the sense of coherence for children’s internalizing problems. This is in support of the resource-potentiating model [59,60] that high levels of maternal warmth and children’s sense of coherence serve as positive conditions that increase an individual’s resources and promote their adaptive development, as manifested in reduced internalizing problems. The current finding provides insights into how to promote Chinese children’s sense of coherence by cultivating mothers’ affection and responsiveness toward their children.

## 5. Limitations and Future Directions

Several limitations of this study should be noted. First, the present longitudinal study had collected only two waves of data, which does not allow us to examine intra-individual patterns of change in sense of coherence and its mediating function over time. Therefore, longitudinal studies with more repeated measures are warranted to obtain a better understanding of the role of the development of a sense of coherence in explaining the pathway from family SES to changes in externalizing and internalizing problems.

Second, self-report data were used and their validity may be threatened by social desirability bias that prompts respondents to answer questions that are viewed favorably by others. Other methods of data collection, such as teacher observation or ratings, should be considered to provide information on a particular behavior in depth and from different perspectives.

Third, children’s subjective SES was not measured in this study. This represents how children perceive their family’s socioeconomic standing relative to others, which is not necessarily comparable to objective SES and a stronger predictor of psychological well-being [29,105]. Therefore, the association between subjective SES and children’s problem behaviors and the role of a sense of coherence as a mechanism underlying the association might be different from the present findings.

Fourth, this study only focused on maternal warmth and it is needed to examine the role of other aspects of maternal parenting (e.g., control) in the association of family SES and problem behaviors. Moreover, a number of studies have suggested that paternal warmth has a pervasive and enduring impact on children’s problem behaviors [106]. Therefore, future research should consider the role of paternal warmth in predicting the longitudinal association between family SES, sense of coherence, and problem behaviors in Chinese children.

Fifth, students’ problem behaviors were reported by the head teacher in the class. Despite teachers being considered reliable informants of children’s problem behaviors, it would be interesting to include reports from peers and parents because problem behaviors occur outside the classroom and in other settings.

Finally, the present study was conducted in a highly urbanized region in China where self-oriented values such as independence and personal uniqueness and social skills such as initiative-taking and self-confidence are highly encouraged [52,107]. On the contrary, rural regions in China have been undergoing less rapid urbanization, and self-confidence and other social skills are less emphasized. Therefore, the functional meaning of the sense of coherence might differ in the two regions. Also, extending the present study to rural children would help researchers and educators identify a possible, common individual (e.g., sense of coherence) and contextual (e.g., maternal warmth) protective resources against problems in both urban and rural children. Moreover, it would be interesting to validate the present findings in more recent samples drawn from urban Chinese children given this study was conducted approximately a decade ago.

Despite these limitations, the present study has some theoretical and practical implications. From a theoretical perspective, in alignment with the Salutogenic model [37,78], the present study extends the literature in suggesting that a sense of coherence is an important mediator in the longitudinal pathways linking family SES to later child adjustment in contemporary China. Moreover, this mediating function of the sense of coherence varied by levels of maternal warmth, suggesting that the mechanism underlying the relation between family SES and child adjustment is contextualized. From a practical perspective, the findings inform mental health interventions of the need of improving the sense of coherence. For example, intervention studies have shown the effectiveness of regular participation in sports and healthy eating in building a strong sense of coherence [108]. Educators and clinicians may use the sense of coherence measures to screen for youth who may benefit from supportive interventions to strengthen their mental health [109]. Parent involvement is a critical component of such interventions in which ways to strengthen parent-child bonding at home to further promote children’s sense of coherence need to be incorporated.

## 6. Conclusions

Overall, this study examined the mediating role of sense of coherence and the moderating role of maternal warmth in the longitudinal relation between family SES and children’s problem behaviors. The results suggested that children’s sense of coherence was the mechanism between family SES and internalizing problem behaviors, but not externalizing problem behaviors. This mediating role was also moderated by maternal warmth and specifically, family SES was negatively associated with internalizing problem behaviors via the sense of coherence for children who perceived high maternal warmth. This study provided evidence of the roles of sense of coherence and maternal warmth in reducing children’s internalizing problem behaviors.

The results indicated that children’s sense of coherence mediated the association between family SES and internalizing problem behaviors, but not externalizing problem behaviors. This mediating role was also moderated by maternal warmth and specifically, family SES was negatively associated with internalizing problem behaviors via the sense of coherence for children who perceived high maternal warmth. Generally, these results highlighted the possible roles of a sense of coherence and maternal warmth in the longitudinal implications of family SES for Chinese children’s internalizing problems.

## Figures and Tables

**Table 1:** Descriptive Statistics for Study Variables.

Variables	1	2	3	4	5	6	7	8	9	10
1 Gender	-									
2 Age	−0.006	-								
3 T1 Family SES	−0.048	−0.267 ***	-							
4 T1 Maternal warmth	−0.068 *	−0.083 *	0.185 ***	-						
5 T1 Sense of coherence	−0.101 **	−0.084 *	0.209 ***	0.431 ***	-					
6 T2 Sense of coherence	−0.082 *	−0.141 ***	0.303 ***	0.370 ***	0.476 ***	-				
7 T1 Externalizing problem behaviors	0.374 ***	−0.018	−0.026	−0.081 *	−0.184 ***	−0.123 **	-			
8. T2 Externalizing problem behaviors	0.341 ***	0.006	−0.037	−0.044	−0.136 **	−0.110 **	0.516 ***	-		
9. T1 Internalizing problem behaviors	−0.125 ***	0.009	−0.037	−0.041	−0.150 ***	−0.105 **	−0.056 ^†^	−0.187 ***	-	
10. T2 Internalizing problem behaviors	−0.056	0.039	−0.043	−0.075 ^†^	−0.147 ***	−0.159 ***	−0.046	0.056	0.225 ***	-
M	-	11.499	0.000	4.049	3.076	3.089	0.000	0.007	0.000	−0.006
SD	-	1.037	1.775	0.968	0.505	0.538	0.985	0.990	0.985	0.970

Note. T1 = Time 1; T2 = Time 2. Gender was dummy-coded (treating girls as the reference group). ^†^ *p* < 0.10, * *p* < 0.05, ** *p* < 0.01, *** *p*< 0.001.

**Table 2:** Mediated Role of Sense of Coherence on Longitudinal Relation Between Family SES and Problem Behaviors.

Outcome Predictor	B	β	SE	95% CI	*t* Value
T2 Sense of coherence					
T1 Sense of coherence	0.48	0.44	0.04	[0.362, 0.516]	11.11 ***
T1 Family SES (a)	0.06	0.19	0.04	[0.11, 0.265]	4.79 ***
T2 Externalizing problem behaviors					
Gender	0.32	0.16	0.04	[0.085, 0.230]	0.01
Age	0.00	0.00	0.04	[−0.074, 0.071]	0.00
T1 Externalizing problem behaviors	0.43	0.43	0.05	[0.338, 0.514]	9.40 ***
T2 Sense of coherence (b)	−0.02	−0.01	0.04	[−0.091, 0.075]	−0.22
T1 Family SES-T2 Sense of coherence (a *b)	−0.00	−0.00	0.01	[−0.018, 0.015]	−0.21
T1 Family SES (c-)	−0.03	−0.05	0.04	[−0.131, 0.026]	−1.31
Total effect (c)	−0.03	−0.05	0.04	[−0.073, 0.012]	−1.41
T2 Internalizing problem behaviors					
Gender	−0.09	−0.05	0.04	[−0.132, 0.031]	−1.14
Age	0.03	0.04	0.04	[−0.051, 0.119]	0.82
T1 Internalizing problem behaviors	0.22	0.22	0.04	[0.126, 0.298]	4.98 ***
T2 Sense of coherence (b)	−0.20	−0.11	0.05	[−0.203, −0.023]	−2.42 *
T1 Family SES-T2 Sense of coherence (a *b)	−0.01	−0.02	0.01	[−0.044, −0.006]	−2.19 *
T1 Family SES (c’)	0.00	0.00	0.05	[−0.092, 0.09]	0.01
Total effect (c)	−0.01	−0.02	0.05	[−0.06, 0.036]	−0.46

*Note.* T1 = Time 1; T2 = Time 2. CI = confidence interval. The coefficients, a, b, and c’, represent direct effects; The coefficient a *b represents an indirect effect. Β for a *b represents the standardized coefficient of an indirect effect.* *p* < 0.05, ** *p* < 0.01, *** *p* < 0.001.

## Data Availability

Data are available through the authors at reasonable request.
